# Intermediate Anthropogenic Disturbances Boost Taxonomic and Phylogenetic Diversities but Reduce Functional Diversity in Subtropical Forests of Eastern China

**DOI:** 10.3390/plants14162529

**Published:** 2025-08-14

**Authors:** Libin Liu, Xiaoyin Guan, Yunquan Wang, Jianhua Chen, Julian Liu, Shuisheng Yu, Zihong Zheng, Mingjian Yu

**Affiliations:** 1 College of Life Sciences, Zhejiang University, Hangzhou 310058, China; liulibin@zjnu.cn (L.L.); yqwang@vip.126.com (Y.W.); 2 College of Life Sciences, Zhejiang Normal University, Jinhua 321004, China; guanxiaoyin@zjnu.edu.cn (X.G.); sky78@zjnu.cn (J.C.); 3 The Administration Center of Zhejiang Jiulong Mountain National Nature Reserve, Suichang 323000, China; ljulian2006@126.com (J.L.); schhc@sina.com (S.Y.)

**Keywords:** species diversity, disturbance history, subtropical forests, ecological restoration

## Abstract

Anthropogenic disturbances significantly impact plant biodiversity in subtropical forests. While prior research has primarily concentrated on taxonomic diversity, other dimensions of biodiversity, such as phylogenetic and functional diversities, remain insufficiently explored. This study simultaneously investigated these three facets of plant diversity in subtropical forests with two distinct disturbance histories in eastern China, aiming to elucidate the effects of intermediate anthropogenic disturbances on biodiversity. Disturbed deciduous broadleaf forests exhibited markedly lower Pielou evenness index values compared to their conserved counterparts (*p* < 0.05). Disturbed evergreen broadleaf forests demonstrated significantly higher species richness, Shannon–Wiener index scores, and phylogenetic diversity relative to those found in conserved forests (*p* < 0.05). Furthermore, both disturbed deciduous broadleaf and mixed evergreen–deciduous broadleaf forests displayed significantly reduced functional richness and quadratic entropy coefficient values when compared with their conserved equivalents (*p* < 0.05). Forest type exerted a significant influence on all three dimensions of biodiversity (*p* < 0.05). In conclusion, intermediate anthropogenic disturbances have the potential to enhance both plant taxonomic and phylogenetic diversities while concurrently diminishing functional diversity within the subtropical forests of eastern China. The mechanisms driving responses in plant diversity to intermediate anthropogenic disturbances vary according to forest types.

## 1. Introduction

Forest disturbance generally refers to the alteration of internal structure, microclimates, biodiversity, and biomass reservoirs, as well as other ecosystem services within a forest ecosystem, while often preserving the integrity of the canopy cover [1,2,3,4,5]. This phenomenon is driven by both natural and anthropogenic factors, with particular emphasis on the latter in recent decades [6,7,8]. Currently, forests all over the world are undergoing significant disturbances, resulting in only approximately one-third of the total forest area being classified as primary forests [9,10,11]. A comprehensive understanding of the impacts of disturbances on forest biodiversity, structure, processes, and functions is crucial for safeguarding primary forests and restoring degraded ones [8,12,13,14].

Biodiversity encompasses the variety and variability of living organisms, along with the ecological complexity inherent in their habitats. It is a product of biological evolution and serves as a fundamental foundation for the sustainable survival of humanity [15,16]. In community ecology, biodiversity is primarily characterized by three key dimensions: taxonomic, phylogenetic, and functional diversities [17,18,19]. Taxonomic diversity, also referred to as species diversity, is a metric that integrates both species richness and their distributional evenness within a community. An increase in taxonomic diversity contributes to enhanced community stability and improved ecosystem functions [15,16]. Phylogenetic diversity pertains to the cumulative phylogenetic distances among species within a community. This dimension is shaped by both the genetic relationships among species and the overall number of species present. Such an understanding provides a more comprehensive perspective on community species diversity from an evolutionary standpoint, thereby elucidating the mechanisms that sustain this diversity [20,21,22,23]. Functional diversity refers to the range of functional traits exhibited by various species within a community. It plays a crucial role in understanding ecosystem processes and their functionalities, as well as responses to environmental changes or disturbances. This concept reflects spatial variation in plant functional traits while offering insights into trait structure, functionality, and resource utilization efficiency within the community [24,25,26,27]. Therefore, these three dimensions of biodiversity represent distinct aspects of plant community assembly processes.

For an extended period, plant ecologists have devoted considerable efforts to investigating the impacts of anthropogenic disturbances on forest plant biodiversity. While considerable progress has been made in understanding taxonomic diversity, there has been comparatively less focus on phylogenetic and functional diversities both before and after disturbances [28,29,30,31]. Consequently, it becomes challenging to fully evaluate the effects of anthropogenic disturbances on overall biodiversity. For instance, some studies have indicated that although anthropogenic disturbances may not significantly alter the numbers of forest species, they can lead to substantial declines in both phylogenetic and functional diversities [32,33]. This finding suggests that even if taxonomic diversity remains stable following anthropogenic disturbances, the potential risk associated with diminished ecosystem functionality cannot be overlooked [31,32,33]. Therefore, a thorough exploration of the effects of anthropogenic disturbances on forest biodiversity, considering taxonomic, phylogenetic, and functional diversities simultaneously, may yield a more nuanced understanding of the relationship between biodiversity and these human-induced changes. Furthermore, this multifaceted analysis enhances our comprehension of the mechanisms underlying forest degradation resulting from anthropogenic perturbations.

The interaction between land and sea, coupled with the uplift of the Qinghai–Tibet Plateau, has endowed East Asia with a monsoonal humid climate system and distinctive zonal evergreen broadleaf forests that exhibit both extensive distribution and remarkable diversity on a global scale. Among these forests, those located in eastern China demonstrate the widest distribution along with the most complex and varied types, constituting a primary component of subtropical evergreen broadleaf forests worldwide [34]. These forests are characterized by their intricate structures and rich species diversity, which not only provide stable ecosystem services but also confer significant environmental benefits [35]. However, frequent anthropogenic disturbances, combined with outdated business philosophies and insufficient understanding of ecological protection, have resulted in primary evergreen broadleaf forests accounting for only 4% of their total distribution area; anthropogenically disturbed forests are now widespread [36]. While some studies have investigated differences in plant species diversity between primary and disturbed forests in this region, they have primarily focused on measuring taxonomic diversity [8,37,38].

In this study, we investigated 48 forests in eastern China, comprising 25 anthropogenically disturbed forests and 23 conserved forests, each characterized by distinct disturbance histories. We conducted a comprehensive examination of the taxonomic, phylogenetic, and functional diversities within these forest ecosystems. Accordingly, we propose the following hypotheses: (1) There are significant differences in taxonomic, phylogenetic, and functional diversities between anthropogenically disturbed forests and conserved forests. (2) Phylogenetic and functional diversities may exhibit different patterns of variation in response to anthropogenic disturbances compared to taxonomic diversity. This study will broaden our understanding of the relationship between biodiversity and anthropogenic disturbances while providing guidance for the ecological restoration of degraded forests in eastern China.

## 2. Results

**Figure 1:**
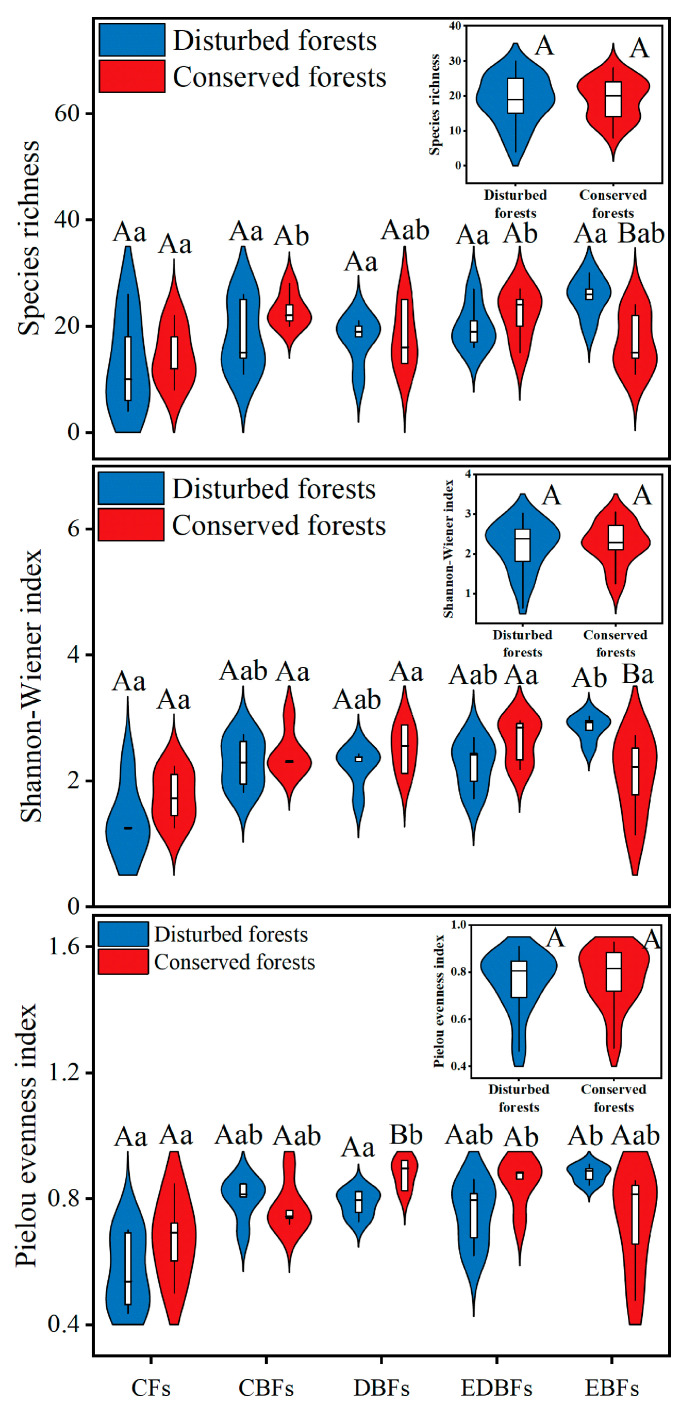
Differences in plant taxonomic diversity between disturbed and conserved forests in eastern China. Capital letters indicate significant differences at the 0.05 level between disturbed and conserved forests of a specific forest type (*t*-test); lowercase letters denote significant differences at the 0.05 level among different forest types within either disturbed or conserved environments (One-Way ANOVA). CFs, coniferous forests; CBFs, mixed coniferous–broadleaf forests; DBFs, deciduous broadleaf forests; EDBFs, mixed evergreen–deciduous broadleaf forests; EBFs, evergreen broadleaf forests.

Generally, disturbed forests exhibited comparable species richness as well as Shannon–Wiener and Pielou evenness indices when compared to conserved forests (Figure 1). Among the various forest types, disturbed deciduous broadleaf forests (DBFs) demonstrated a significantly lower Pielou evenness index (*t* = −2.946, *p* = 0.026) than their conserved counterparts, while disturbed evergreen broadleaf forests (EBFs) showed significantly higher species richness (*t* = 2.832, *p* = 0.022) and Shannon–Wiener index (*t* = 2.606, *p* = 0.031) in comparison to those of the conserved forests (Figure 1). Forest type had a significant impact on taxonomic diversity (Figure 1). In disturbed environments, coniferous forests (CFs) exhibited a markedly lower Shannon–Wiener index (*F* = 9.421, *p* = 0.027) relative to EBFs (Figure 1). In conserved environments, CFs displayed a significant reduction in species richness compared to mixed coniferous–broadleaf forests (CBFs; *F* = 2.535, *p* = 0.014) and mixed evergreen–deciduous broadleaf forests (EDBFs; *F* = 2.535, *p* = 0.025; Figure 1). CFs revealed a notably lower Pielou evenness index when contrasted with DBFs (*F* = 2.596, *p* = 0.020) and EDBFs (*F* = 2.596, *p* = 0.018; Figure 1). Furthermore, the interaction effect of anthropogenic disturbances and forest type exerted significant effects on both Shannon–Wiener (*F* = 3.422, *p* = 0.017) and Pielou evenness (*F* = 3.299, *p* = 0.020) indices.

**Figure 2:**
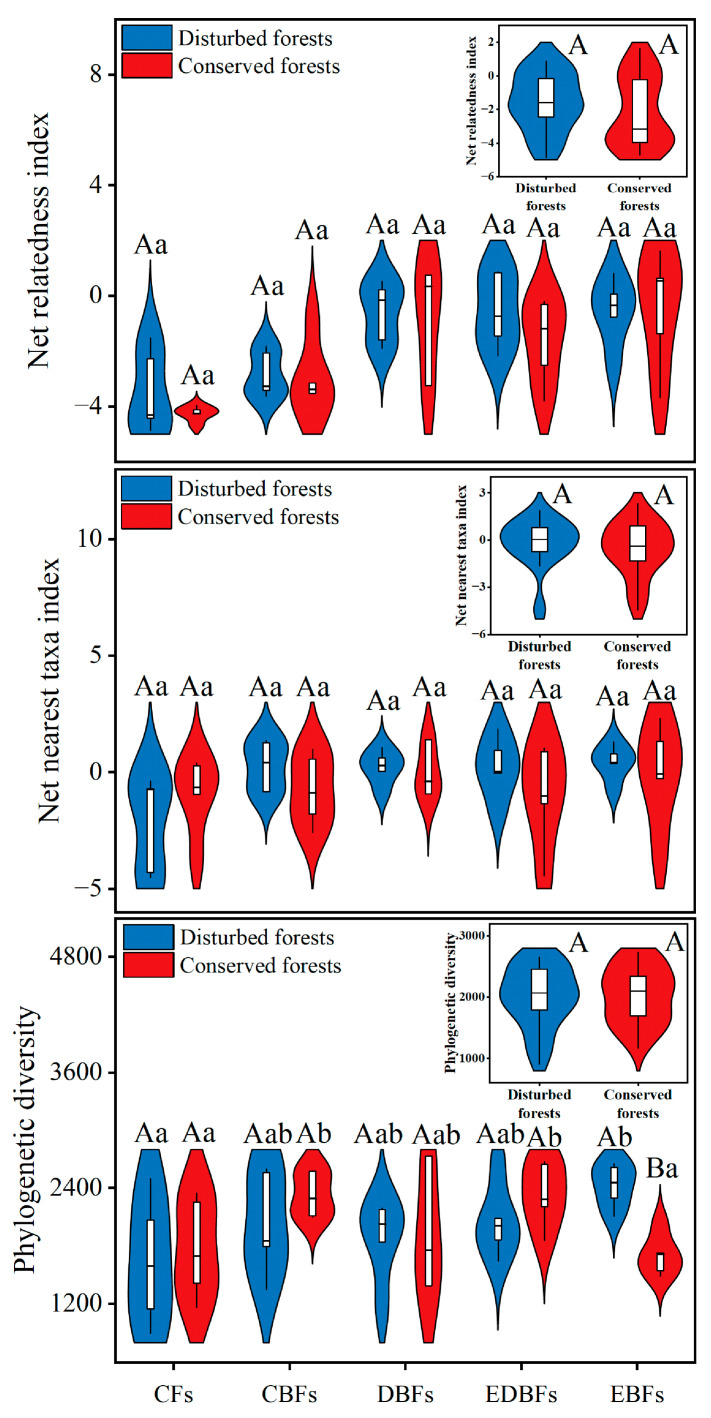
Differences in plant phylogenetic diversity between disturbed and conserved forests in eastern China. Capital letters indicate significant differences at the 0.05 level between disturbed and conserved forests of a specific forest type (*t*-test); lowercase letters denote significant differences at the 0.05 level among different forest types within either disturbed or conserved environments (One-Way ANOVA). CFs, coniferous forests; CBFs, mixed coniferous–broadleaf forests; DBFs, deciduous broadleaf forests; EDBFs, mixed evergreen–deciduous broadleaf forests; EBFs, evergreen broadleaf forests.

Similar to taxonomic diversity trends observed earlier, disturbed forests generally exhibited comparable values of net relatedness index (NRI), net nearest taxa index (NTI), and phylogenetic diversity (PD) when compared to conserved forests. For different forest types within the disturbed category, PD values of EBFs were significantly higher than those of their conserved counterparts (*t* = 5.200, *p* < 0.001; Figure 2). Forest type exclusively influenced PD in both disturbed and conserved environments (Figure 2). In disturbed settings, CFs demonstrated significantly lower PD values compared to EBFs (*F* = 1.894, *p* = 0.015). In conserved habitats, both CFs (*F* = 2.791, *p_EDBFs_* = 0.040, *p_CBFs_* = 0.041) and EBFs (*p_EDBFs_* = 0.023, *p_CBFs_* = 0.023) exhibited markedly reduced PD values relative to EDBFs and CBFs (Figure 2). However, the interaction effect of anthropogenic disturbances and forest type did not significantly impact forest phylogenetic diversity.

**Figure 3:**
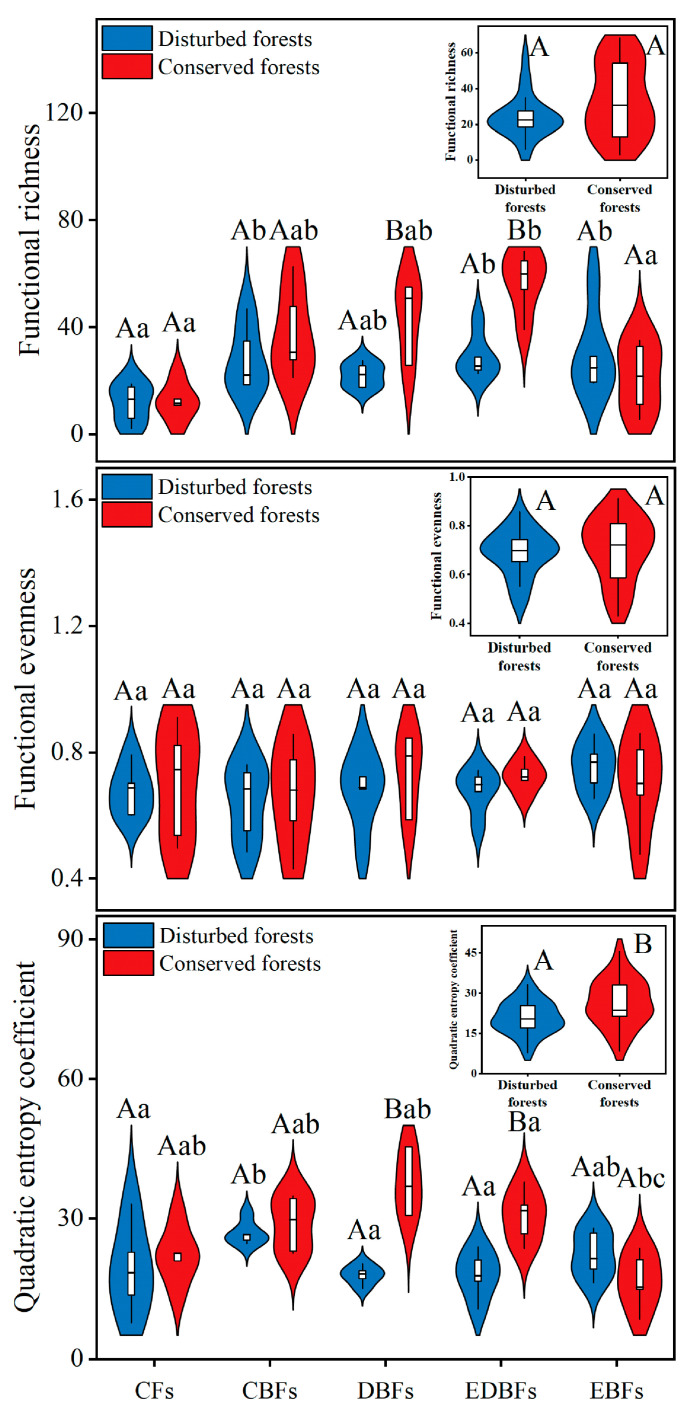
Differences in plant functional diversity between disturbed and conserved forests in eastern China. Capital letters indicate significant differences at the 0.05 level between disturbed and conserved forests of a specific forest type (*t*-test); lowercase letters denote significant differences at the 0.05 level among different forest types within either disturbed or conserved environments (One-Way ANOVA). CFs, coniferous forests; CBFs, mixed coniferous–broadleaf forests; DBFs, deciduous broadleaf forests; EDBFs, mixed evergreen–deciduous broadleaf forests; EBFs, evergreen broadleaf forests.

In contrast to the aforementioned two dimensions of biodiversity, functional diversity is more profoundly affected by anthropogenic disturbances. This effect is particularly evident in DBFs (*t_FRic_* = −3.023, *p* = 0.023; *t_RaoQ_* = −5.922, *p* < 0.001) and EDBFs (*t_FRic_* = −4.545, *p* = 0.002; *t_RaoQ_* = −3.769, *p* = 0.005); these two forest types presented significantly lower functional richness (FRic) and quadratic entropy coefficient (RaoQ) values in disturbed environments compared to those found in conserved settings (Figure 3). Forest type exclusively influenced FRic and RaoQ within both disturbed and conserved environments (Figure 3). In disturbed environments, CFs demonstrated significantly lower FRic values compared to EBFs (*F* = 2.644, *p* = 0.011) as well as EDBFs (*p* = 0.019) and CBFs (*p* = 0.022), while CBFs exhibited significantly higher RaoQ values than CFs (*F* = 2.403, *p* = 0.038) along with DBFs (*p* = 0.018) and EDBFs (*p* = 0.019). In conserved environments, EDBFs showed markedly higher FRic (*F* = 9.348, *p* = 0.017) and RaoQ (*F* = 7.236, *p* = 0.049) values than EBFs; they also displayed a notably higher FRic value (*p* = 0.002) when compared to CFs (Figure 3). Moreover, the interaction effect of anthropogenic disturbances and forest type also had significant impacts on both FRic (*F* = 4.043, *p* = 0.008) and RaoQ (*F* = 6.468, *p* < 0.001) indices.

## 3. Discussion

Few studies have examined the effects of anthropogenic disturbances on plant biodiversity in the subtropical forests of China, with the majority of existing research primarily concentrating on taxonomic diversity [8,37,38]. Phylogenetic and functional diversities, particularly the latter, remain insufficiently explored [39,40]. This study simultaneously investigated these three dimensions of biodiversity, thereby offering a comprehensive assessment of changes in plant diversity before and after anthropogenic disturbances in the subtropical forests of eastern China. Such an investigation aspires to bridge gaps in our understanding of the intricate relationship between biodiversity and anthropogenic disturbances within this ecological framework. However, it is noteworthy that only woody individuals with a D ≥ 5 cm were incorporated into this analysis; future research should also consider understory saplings, seedlings, lianas, shrubs, and herbs for a more holistic perspective.

Among the myriad models proposed to elucidate the relationship between taxonomic diversity and anthropogenic disturbances, the intermediate disturbance hypothesis stands out as particularly significant. This hypothesis posits that taxonomic diversity reaches its zenith at intermediate levels of disturbance, both in terms of duration (where the disturbance interval is approximately half the average lifespan of the dominant species) and intensity (where such intensity leads to either a mortality rate of around fifty percent or a substantial alteration in half of the community structure or resource utilization rates) [41,42,43]. In this study, intermediately disturbed EBFs demonstrated significantly higher species richness and Shannon–Wiener index compared to their conserved counterparts, which aligns with the predictions of the intermediate disturbance hypothesis. Conversely, for other forest types, disturbed and conserved forests displayed comparable indices of taxonomic diversity, thereby challenging the expectations established by this hypothesis. The mechanisms underlying taxonomic diversity in response to disturbances are far more intricate than those suggested by this hypothesis, varying according to species groups and forest types [8,44].

From the perspective of phylogenetic diversity, our analysis revealed that both intermediately disturbed and conserved forests exhibited negative values for NRI and NTI. The observed divergences in the phylogenetic structures of these forest communities indicate that they are composed of species with distant genetic relationships; competitive exclusion emerges as the primary factor influencing forest community assembly [22]. While the NRI and NTI values for conserved forests are more negative than those for disturbed forests, this suggests that species within conserved forests are more distantly related to one another. This conclusion is further supported by the larger PD value observed in these forests. Due to higher species richness and the Shannon–Wiener index, the PD values of disturbed EBFs are also significantly greater than those of their conserved counterparts. From the standpoint of functional diversity, our findings demonstrate that anthropogenic disturbances have markedly diminished both FRic and RaoQ values in BDFs and EDBFs. This decline indicates that anthropogenic disturbances may exacerbate competition among plant species while concurrently undermining their efficiency in resource utilization, thereby detrimentally impacting forest ecosystem functions [8,45,46].

Therefore, drawing upon our comprehensive analysis of the three dimensions of biodiversity, we assert that intermediate anthropogenic disturbances have the potential to enhance both plant taxonomic and phylogenetic diversities within the subtropical forests of eastern China. Nevertheless, it is crucial to acknowledge that these disturbances may concurrently result in a reduction of plant functional diversity across these forest ecosystems [32,33,47].

Due to extensive anthropogenic disturbances, the majority of primary forests in eastern China have been transformed into degraded ecosystems, including grasslands, shrublands, and secondary forests. Consequently, restoring these degraded vegetative areas has emerged as a significant challenge in eastern China [36]. The enhancement of taxonomic diversity is frequently employed as an indicator for assessing the success of restoration efforts [8,28,48]. Concurrently considering taxonomic, phylogenetic, and functional diversities offers an additional avenue for predicting the effectiveness of restoration initiatives and the potential for local vegetation recovery [47,49]. In this study, we found that the taxonomic and phylogenetic diversities of disturbed forests have recovered to levels comparable to those of conserved forests; however, the functional diversity within disturbed forests remains significantly lower than that observed in conserved forests. Ecosystem functions related to biodiversity explored in this study—alongside others such as water conservation and carbon sequestration—are still considerably distant from those characteristic of primary forests. Therefore, a comprehensive exploration of biodiversity within both disturbed and conserved forest ecosystems is crucial not only for providing data to benchmark global and regional biodiversity models [50,51] but also for facilitating regional biodiversity inventories and vegetation restoration. Protecting the remaining primary forests while recovering degraded vegetation could potentially mitigate climate change impacts, sustain regional environmental health, and enhance livelihoods for local communities in eastern China.

## 4. Materials and Methods

### 4.1. Study Area

**Figure 4:**
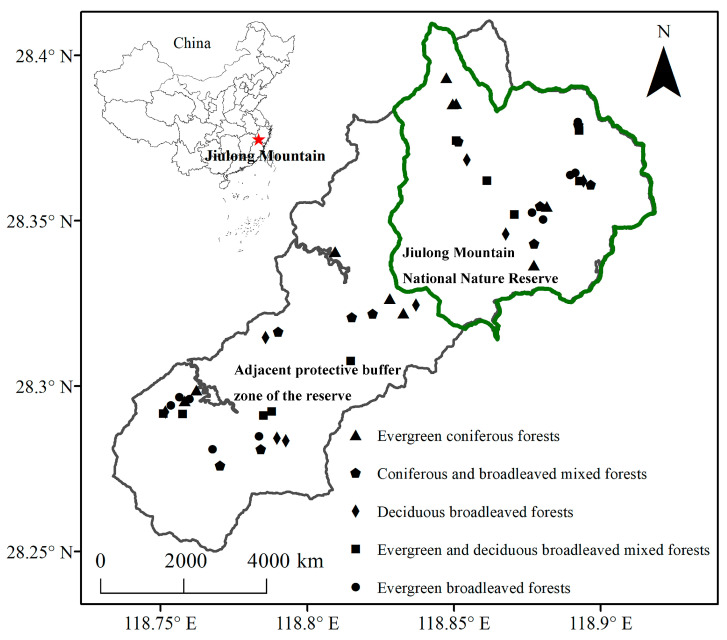
Locations of sampling plots within the Jiulong Mountain National Nature Reserve and its adjacent protective buffer zone in eastern China.

The Jiulong Mountain National Nature Reserve (118°49′–118°55′ E, 28°19′–28°24′ N), covering a total area of 55.25 km^2^, is located in Suichang County in the southwestern part of Zhejiang Province, eastern China (Figure 4). This reserve occupies a strategically significant position at the intersection of Zhejiang, Fujian, and Jiangxi provinces and has been recognized as one of the 25 global priority areas and one of the 35 pivotal regions in China for biodiversity conservation [52,53]. The area is characterized by a mid-subtropical climate that is profoundly influenced by humid monsoons. The mean annual temperature ranges from 8.0 to 14.5 °C; January witnesses the lowest monthly average temperatures between 0 and 3.8 °C, while July records the highest monthly average, ranging from 18.0 to an impressive maximum of 25.0 °C. Extreme recorded temperatures have reached up to a maximum of 37.0 °C and dropped to a minimum of −10.5 °C, respectively. Growing degree days calculated with a threshold of 10 °C vary widely between approximately 2500 and an astonishingly high figure reaching up to about 4700 °C. Annual precipitation averages within an ample range from around 1600 mm upwards to over 2000 mm, with annual sunshine duration averaging approximately an impressive total of about 1925 h [8]. The geological foundation primarily consists of granitic porphyry, rhyolitic porphyry, welded tuff, and altered acidic volcanic rocks, along with certain syenitic porphyries derived from Jurassic volcanic lava and pyroclastic deposits. The predominant soil type found throughout this reserve is classified as mountainous red-yellow soil [54]. The dominant vegetation within this sanctuary manifests predominantly as zonal EBF; however, various other forest types are interspersed throughout, including EDBF, DBF, CF, and CBF [8].

### 4.2. History of Sampling Sites and Vegetation Survey

Field measurements were meticulously conducted within the Jiulong Mountain National Nature Reserve and its adjacent protective buffer zone (Figure 4). The reserve is distinguished by its well-conserved native vegetation, which provides a crucial habitat for numerous endangered and rare animal and plant species, largely due to its remote location, high elevation, and restricted accessibility for transportation. Initially established as a provincial reserve in 1983, it achieved the prestigious designation of National Nature Reserve in 2003. Over the past four decades, various disturbances such as fire, logging activities, firewood collection, and livestock grazing have been stringently regulated by the administration center within the reserve. In contrast, while rigorous fire control measures have also been diligently enforced in the adjacent protective buffer zone, intermediate anthropogenic disturbances—including logging, firewood collection, and livestock grazing—continue to occur sporadically [8].

After conducting a comprehensive on-site survey of the vegetation, which included an analysis of their distribution, dominant species, interior habitats, and disturbance histories, five distinct forest types previously identified were selected from within the reserve and its adjacent protective buffer zone. Deciduous broadleaf forests are limited in their distribution within the reserve, which presents challenges in selecting a sufficient number of representative plots (each measuring 20 m × 20 m, as determined by the minimum area method) for this forest type. As a result, only three plots were established for this specific category. In contrast, five plots were designated for each of the other four forest types, both within the reserve and its adjacent protective buffer zone [55,56]. Consequently, a total of 48 plots were meticulously delineated (Figure 4). The plots were independent, as the distance between plots of the same forest type was maintained at greater than 100 m. Comprehensive data was recorded for each plot, including geographical coordinates, elevation, slope gradient, aspect orientation, and coverage of exposed rock outcrops. All woody plants with a diameter at breast height (D) ≥ 5 cm were thoroughly documented along with their species identity, D measurements (obtained using a diameter tape), height assessments (estimated by an experienced individual), and canopy width dimensions (canopy projection width, measured with a steel tape).

### 4.3. Functional Trait Measurements

In each forest plot, the dominant species—identified as those exhibiting the highest biomass—that constituted over 90% of the total forest biomass were meticulously selected for functional trait measurements. The biomass of all plant individuals was estimated using established biomass allometric models [8]. From each plot, five healthy dominant individuals per species were chosen. Four branches were harvested from distinct positions on the sunlit side of the tree canopy utilizing an averruncator. From each branch, five mature broad leaves or ten mature needle leaves and a twig aged two to three years (approximately 20 cm in length) were sampled. In total, either 20 or 40 leaves and four twigs were collected from each individual. Additionally, three bark samples and three stem samples were procured from each individual at the breast height position employing a sickle and an increment borer. Each sample was placed in a dry and sterile preservation bag to ensure optimal conditions for subsequent analyses.

Leaf area (LA) was meticulously quantified utilizing the WinFOLIA multipurpose leaf area meter (Regent Instruments, Québec City, QC, Canada). The measurements of leaf thickness (LT) and bark thickness were accurately determined with an electronic vernier caliper, which boasts a precision of 0.01 mm. Each sample’s fresh weight was recorded using a highly sensitive electronic balance with a resolution of 0.001 g. The volume of leaf samples was calculated by multiplying LA and LT, while the volumes for twig, bark, and stem samples were assessed employing the drainage method [8]. Leaf samples underwent desiccation at 80 °C for a duration of 48 h; in contrast, twig, bark, and stem samples were dried for 72 h to ensure accurate determination of their dry weights. Specific leaf area, leaf tissue density, leaf dry matter content, twig tissue density, twig dry matter content, bark tissue density, bark dry matter content, stem tissue density, and stem dry matter content values were computed in accordance with established methodologies as outlined by Cornelissen et al. in 2003 [57] and Pérez-Harguindeguy et al. in 2013 [58].

After conducting measurements of morphological traits, all samples were finely ground into a powder and subsequently sieved through a 0.2 mm mesh. The total carbon and total nitrogen contents in the leaf, twig, bark, and stem were analyzed using the Vario MACRO Cube (Thermo Scientific, Bremen, Germany). Additionally, the total phosphorus contents for the leaf, twig, bark, and stem were determined employing the iCAP 6300 ICP-OES Spectrometer Analyzer (Thermo Scientific, Waltham, MA, USA). Furthermore, we calculated the carbon-to-nitrogen ratios, carbon-to-phosphorus ratios, as well as nitrogen-to-phosphorus ratios for each component: leaf, twig, bark, and stem.

### 4.4. Data Analysis

Taxonomic diversity was evaluated through the assessment of species richness, the Shannon–Wiener index, and the Pielou evenness index. Phylogenetic diversity was quantified using the NRI, NTI, and PD. Functional diversity was examined by employing FRic, functional evenness (FEve), and RaoQ. The formulae for these nine diversity indices were described in detail in Table 1 [22,25,59,60,61]. Prior to further statistical analyses, biodiversity index data (Table S1) were tested for normality; results indicated that the data conformed to a normal distribution. A two-way analysis of variance (ANOVA) was conducted to evaluate the interaction effects of anthropogenic disturbances and forest type on biodiversity indices. An independent samples *t*-test was performed to assess differences in biodiversity indices between disturbed and conserved forests. Additionally, a one-way ANOVA was utilized to analyze variations in biodiversity indices among different forest types. Post hoc tests were conducted using Fisher’s least significant difference method for homogeneity of variance or Tamhane’s method for heterogeneity of variance following the homogeneity test results. All calculations related to biodiversity indices and statistical procedures were performed using R software version 4.1.3 [62].

## 5. Conclusions

Overall, our investigation into the taxonomic, phylogenetic, and functional diversities of plants reveals that intermediate anthropogenic disturbances can enhance both plant taxonomic and phylogenetic diversities while reducing functional diversity in the subtropical forests of eastern China. The mechanisms governing these responses vary significantly depending on the specific forest types involved. A better understanding of how anthropogenic disturbances affect forest biodiversity will aid in more effective conservation and restoration efforts in eastern China.

## Figures and Tables

**Table 1:** Plant diversity indices and their calculations in subtropical forests of eastern China.

Index	Calculation	Meaning of Terms
Species richness (R)	R=S	S is the total number of species P_i_ is the proportion of total abundance of the ith species i is a species within the community
Shannon–Wiener index (H)	$H=-\sum_{i=1}^{S}P_{i}\ln P_{i}$	
Pielou evenness index (E)	$E=\frac{H}{\ln S}$	
Net relatedness index (NRI)	$NRI=-1\times \frac{MPD_{s}-MPD_{mds}}{SD(MPD_{mds})}$	MPD_s_ and MNTD_s_ are the observed mean pairwise distance (MPD) values and the mean nearest taxon distance (MNTD) values, respectively MPD_mds_ and MNTD_mds_ are the simulated MPD values and MNTD values, respectively, which are derived from 999 random simulations conducted under the null model SD is the standard deviation C is the sum of shortest path branch lengths of phylogenetic trees composed of all species in the study area c is a branch of C Lc is the branch length of c
Net nearest taxa index (NTI)	$NTI=-1\times \frac{MNTD_{s}-MNTD_{mds}}{SD(MPD_{mds})}$	
Phylogenetic diversity (PD)	$PD=\sum_{\left\{c\in C\right\}}Lc$	
Functional richness (FRic)	$FRic=\frac{SF_{ic}}{R_{c}}$	SF_ic_ is the niche space occupied by species within the community R_c_ is the absolute range of trait c S is the total number of species PEW_i_ is the weighted uniformity of specie i d_ij_ is the overlap of the probability density function of trait for species i and j P_i_ and P_j_ are the proportion of the individuals of species i and species j to the total number of species within the community
Functional evenness (FEve)	$FEve=\frac{\sum_{i=1}^{S-1}\min(PEW_{i}\frac{1}{S-1})-\frac{1}{S-1}}{1-\frac{1}{S-1}}$	
Quadratic entropy coefficient (RaoQ)	$RaoQ=\sum_{i=1}^{S-1}\sum_{j=i+1}^{S}d_{ij}P_{i}P_{j}$	

## Data Availability

The original contributions presented in this study are included in the article/Supplementary Materials. Further inquiries can be directed to the corresponding author.

## Supplementary Materials

The following supporting information can be downloaded at https://www.mdpi.com/article/10.3390/plants14162529/s1.
